# Vitamin B_6_: A Molecule for Human Health?

**DOI:** 10.3390/molecules15010442

**Published:** 2010-01-20

**Authors:** Hanjo Hellmann, Sutton Mooney

**Affiliations:** Washington State University, Abelson 435, P.O. Box 66224, Pullman, WA, USA

**Keywords:** vitamin B_6_, PDX, de novo, salvage, health

## Abstract

Vitamin B_6_ is an intriguing molecule that is involved in a wide range of metabolic, physiological and developmental processes. Based on its water solubility and high reactivity when phosphorylated, it is a suitable co-factor for many biochemical processes. Furthermore the vitamin is a potent antioxidant, rivaling carotenoids or tocopherols in its ability to quench reactive oxygen species. It is therefore not surprising that the vitamin is essential and unquestionably important for the cellular metabolism and well-being of all living organisms. The review briefly summarizes the biosynthetic pathways of vitamin B_6_ in pro- and eukaryotes and its diverse roles in enzymatic reactions. Finally, because in recent years the vitamin has often been considered beneficial for human health, the review will also sum up and critically reflect on current knowledge how human health can profit from vitamin B_6_.

## 1. Introduction

The B vitamins are a group of water soluble, chemically quite distinct compounds to which other than vitamin B_6_, vitamin B_1_ (thiamine), B_2_ (riboflavin), B_3_ (niacin or niacin amide), B_5_ (pantothenic acid), B_7_ (biotin), B_9_ (folic acid), and B_12_ (various cobalamins) also belong [1]. Historically, it was believed that only one vitamin B existed with a critical function for maintenance of growth and health and prevention of characteristic skin lesions in animals and human [2]. However, with ongoing research it became obvious that vitamin B actually comprised a group of compounds that was collectively called the ‘vitamin B complex’.

**Scheme 1:**
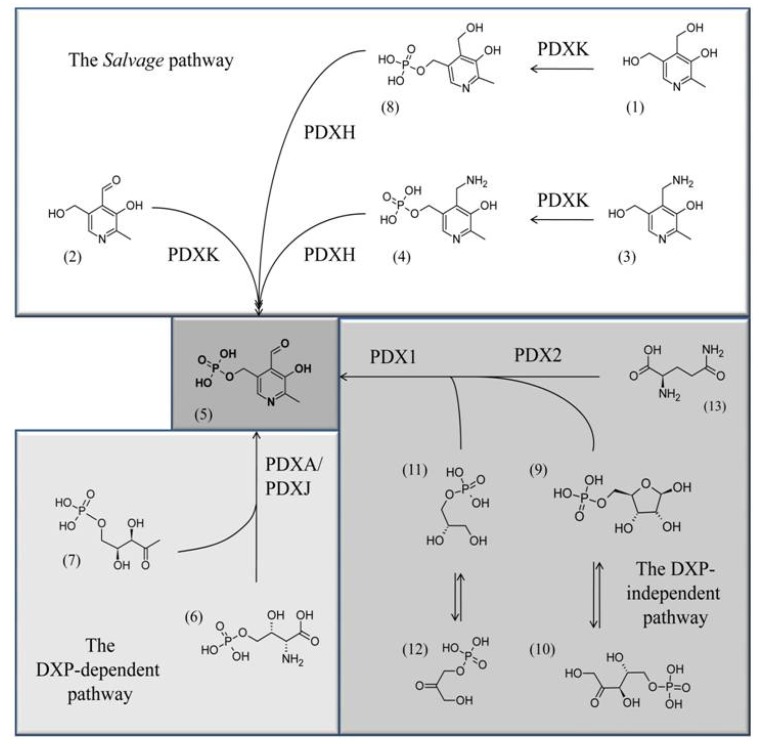
The three known pathways for PLP biosynthesis: one *salvage* pathway, and two *de novo* pathways, a DXP-dependent one and a DXP-independent one. Chemical structures: (**5**) PLP; (**7**) deoxyxylulose 5’-phosphate, (**6**) 4-(phosphohydroxy)-L-threonine; (**11**) glyceraldehyde 3’-phosphate; (**12**) dihydroxyacetone phosphate; **(9)** ribose 5’-phosphate; (**10**) ribulose 5’-phosphate, (**13)** glutamine, (**3**) PM, (**4**) PMP, (**1**) PN, (**8**) PNP, (**2**) PL.

Vitamin B_6_ (vitB_6_ from here on) itself is an enzymatic co-factor required for more than 140 biochemical reactions including transaminations, aldol cleavages, α-decarboxylations, racemizations, β- and γ- eliminations, and replacement reactions. Most of these reactions are related to amino acid biosynthesis and degradation, but vitB_6_ is also involved in other processes including sugar and fatty acid metabolism [3]. It comprises a set of three different pyridine derivatives called pyridoxine (PN; **1**), pyridoxal (PL; **2**), and pyridoxamine (PM; **3**). They differ in a variable group present at their 4-position with PN carrying a hydroxymethyl group, and PL (**2**) and PM (**3**) having an aldehyde and an aminomethyl group, respectively. Furthermore, all three B_6_ vitamers are phosphorylated by a kinase, which is a requirement for their role as cofactors in enzymatic reactions (Scheme 1). While pyridoxamine-5’-phosphate (PMP; **4**) has been reported to function as a co-factor, it is pyridoxal 5’-phosphate (PLP; **5**) that is the biologically most active form [4,5].

A growing number of interesting and helpful new resources have been established in the last years that focus primarily on vitB_6_ related issues. For example, an online database has been launched that allows searching whole genomes for PLP-dependent enzymes, and which also provides information on critical aspects such as the biochemical pathways requiring PLP (**5**) and the classification of PLP-dependent enzymes (http://bioinformatics.unipr.it/cgi-bin/bioinformatics/B6db/home.pl) [3]. In addition, a database has been established that allows searching for mutated PLP-dependent enzymes in various organisms (http://www.studiofmp.com/plpmdb/home.htm) [6].

## 2. Suggested Reaction Mechanisms of VitB_6_ for Amino Acid Metabolism

In most cases PLP (**5**) is covalently bound to the ε-amino group of a conserved lysine residue in the active center of a PLP-dependent enzyme, with its 5’-phosphate group being buried in a conserved phosphate-binding cup [7]. It is suggested that reactions are initiated by the formation of a geminal diamine intermediate between the aldehydic carbon atom of PLP (**5**) and an amino group of the substrate. This is followed by its rapid breakdown and the formation of an external aldimine (Schiff base) between PLP (**5**) and the substrate causing the release of the lysine residue of the enzyme from PLP (**5**). From this point on subsequent reactions mainly depend on the specific, participating enzymes that guide and modulate the next steps leading to e.g. racemisations, β- and γ- eliminations.

## 3. Three Different Biosynthetic Pathways for VitB_6_ Are Known

Three different pathways for vitB_6_ biosynthesis have been described which will be just briefly summarized, as they were topics of other recent reviews [8,9]. In eubacteria like *Escherichia coli*, the vitamin can be *de novo* synthesized by the concerted activities of the pyridoxine biosynthesis proteins A and J (PdxA (EC 1.1.1.262) and PdxJ (EC 2.6.99.2), respectively) which use 4-phospohydroxy-L-threonine (4HPT; **6**) and deoxyxylose 5’-phosphate (DXP; **7**) to synthesize pyridoxine 5’-phosphate (PNP; **8**) (Scheme 1) [10,11,12]. In bacteria, archaea, and eukarya a second *de novo* pathway is known that synthesizes PLP (**5**) from ribose 5’-phosphate (**9**) or ribulose 5’-phosphate (**10**), in combination with either glyceraldehyde 3’-phosphate (**11**) or dihydroxyacetone phosphate (**12**) and glutamine (**13**) (Scheme 1) [13,14,15,16,17].

Here two pyridoxine biosynthetic enzymes (PDX) are active: while PDX2 functions as a glutaminase that deaminates glutamine to glutamate in order to supply nitrogen for the PLP heterocycle, PDX1 arranges the final ring closure [18,19,20,21,22,23,24]. Because of a different sugar precursor used for the biosynthesis of the vitamin, the *de novo* pathway from eubacteria is known as the DXP-dependent pathway, while the other is the DXP-independent pathway [14]. In addition to the two *de novo* pathways, most organisms also have a *salvage* pathway that converts the different B_6_ vitamers to PLP (**5**). This is achieved by the concerted activities of an oxidase, PDXH (EC 1.4.3.5), and a kinase, PDXK (EC 2.7.1.35) (Scheme 1) [8,25]. Most animal organisms, including humans, have a *salvage* pathway, however, they lack the enzymatic machinery for *de novo* synthesis and rely on external uptake of the vitamin from food [16].

## 4. VitB_6_ and Its Healthy Face

Since its discovery in 1932 by the Japanese scientist S. Ohdake, vitB_6_ has been discussed in relationship to health issues [26]. In these early works from, for example, Ohdake or the Hungarian scientist P. Györgi, vitB_6_ was associated with pellagra, a skin disease that is based on multi-vitamin deficiencies that mostly occurs in context with niacin undersupply [27,28,29]. A search through the public literature data basis (http://www.ncbi.nlm.nih.gov/) for health aspects associated with vitB_6_ yields a surprisingly high number of articles (>900). Furthermore, the current *Recommended Dietary Allowance* per day by the National Institute of Health (NIH) of the USA is around 2 mg with an upward tolerance of 100 mg per day for adults. A recent U.S. study, which tested the blood PLP levels in around 8,000 patients, demonstrated a widespread deficiency of the vitamin among all tested subgroups, and the authors suggested an increase of the daily allowance from around 2 mg to 3 to 4.9 mg per day [30]. It has been reported for animal models, that continuous uptake of very high doses (e.g. 400 mg/kg) can lead to peripheral sensory neuropathy and nerve degeneration [31,32]. These problems are generally reversible when supplementation is stopped. Additionally some studies have suggested that increased levels of the B_6_ vitamers and some derivatives can generate toxic photoproducts as a result of UV irradiation [33,34,35]. However, the applied daily dosages were far beyond any physiological concentrations an organism is normally exposed to, making it unlikely that such vitB_6_ induced impacts will be observed. Because of the great interest in vitB_6_ as a therapeutic and pharmaceutical compound, its reactive capability, and its potent antioxidative characteristics, we summarize in the following paragraphs some of the relevant topics related to these issues.

### 4.1. Therapeutic applications by using drugs against PLP-dependent enzymes

PLP-dependent enzymes are highly diverse and the reactions they facilitate are estimated to represent 4% of all known catalytic activities; hence, many of them are being explored as targets for therapeutic agents (for an excellent overview see [5]). We chose three major examples for this review to illustrate the potentials of this approach in disease control: malaria, sleeping sickness, and cancer treatment.

**Scheme 2:**
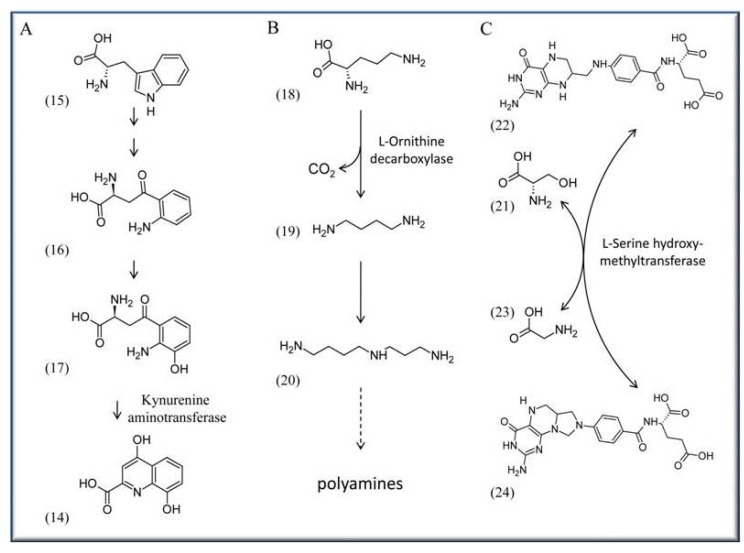
Enzymatic reaction that are targets for pharmaceutical approaches. Shown are only the PLP-dependent enzymes. (A) Synthesis of xanthurenic acid (**14**) from L-tryptophan (**15**), *via* the intermediates L-kynurenine (**16**) and 3-hydroxykynurenine (**17**). (B) Synthesis of putrescine (**19**) from L-ornithine (**18**), leading subsequently to the synthesis of spermidine (**20**) and other polyamines. (C) Synthesis of tetrahydrofolate (**22**) and serine (**21**) to glycine (**23**) and 5,10-methylenetetrahydrofolate (**24**). *Note*: in this and the next Schemes only the PLP-dependent enzymes are shown.

One of the most threatening human diseases is malaria, with more than 300-500 million infected people worldwide and an annual death toll of up to one million people (http://www.unicef.org/health/ index_malaria.html). Several approaches are currently underway in an effort to affect the life cycle or metabolism of the pathogen *Plasmodium falciparum*, the cause of malaria. One such approach is to impair biosynthesis of xanthurenic acid (**14**), which is essential for gametogenesis and fertility of the pathogen [36,37,38]. The acid is synthesized as part of the L-tryptophan (**15**) degradation pathway from L-kynurenine (**16**) *via* 3-hydroxykynurenine (**17**) by the activity of the PLP-dependent kynurenine aminotransferase (EC 2.6.1.7) [39] (Scheme 2A).

A possible strategy involves developing specific drugs that reduce activity of the aminotransferase. This might lower the levels of 3-hydroxykynurenine (**17**) in *P. falciparum* infected mosquitoes potentially reducing or even preventing malaria transmission to humans. A similar direction was recently proposed by channeling synthetic pyridoxyl-amino acid adducts into the pathogen, which can phosphorylate these compounds mediated by PDXK kinase [40]. After binding by a PLP-dependent enzyme, such phosphorylated compounds should inhibit these enzymes and affect further metabolism. Müller and co-workers successfully tried pyridoxyl-tryptophan methyl ester to inhibit proliferation of *P. falciparum* opening up the possibility for a novel malaria treatment in the future [40]. Because *P. falciparum* expresses PDX1/PDX2 proteins, which humans lack, a potential approach can also be to target these *de novo* pathway proteins by specific drugs [41]. However, no specific approach has been reported so far.

African sleeping sickness is another severe epidemic disease with an estimated 300–500 thousand people affected in various African countries (http://www.sbri.org/diseases/african.asp). It is caused by the protist *Trypanosoma brucei* and transmitted by flies of the Genus *Glossina.* A target to treat sleeping sickness in affected patients is the PLP-dependent enzyme ornithine decarboxylase (ODC; E.C. 4.1.1.17). It catalyzes the step from L-ornithine (**18**) to the diamine putrescine (**19**), an initial step in the production of polyamines (Scheme 2B). α-Difluoromethylornithine (DFMO) is a proven irreversible inhibitor of ODC activity and works by forming a covalent bond with a cysteine residue of ODC after decarboxylation [42,43,44]. Although DFMO is an approved drug in treating sleeping sickness caused by *T. brucei*, the precise reason for its effectiveness is not fully resolved because human and *T. brucei* ODCs are comparably affected by the agent [45]. It is suggested that this effect is based on metabolic differences: a more rapid turnover of the host’s ODC on the one side, and on the other side *T. brucei*’s high demand for the synthesis of the polyamine trypanothione, a specific dithiol essential for the detoxification system of Trypanosomes and Leishmania parasites [46,47].

Targeting PLP-dependent enzymes is also discussed in context with cancer. Here an interesting candidate is, for example, serine hydroxymethyltransferase (SHMT; EC 2.1.2.1), which catalyzes the reversible transfer of the Cβ of serine (**21**) to tetrahydrofolate (**22**) to form glycine (**23**) and 5,10-methylenetetrahydrofolate (**24**) (Scheme 2C). Because of 5,10-methylenetetrahydrofolate (**24**), which serves as a methyl donor in many reactions, SHMT activity is critical for one-carbon metabolism, the biosynthesis of methionine, lipids, formyl-tRNA and pyrimidine. The latter is of special interest as apparently SHMT activity is coupled to some extent with increased demand for DNA biosynthesis. For example there is evidence in tumors with highly proliferating, mitotically active cells, that serine is preferentially channeled for DNA biosynthesis [48,49]. Consequently SHMT is a proposed target in developing drugs for chemotherapy [50,51].

### 4.2. VitB_6_ in context with cardiovascular disease and blood pressure

**Scheme 3:**
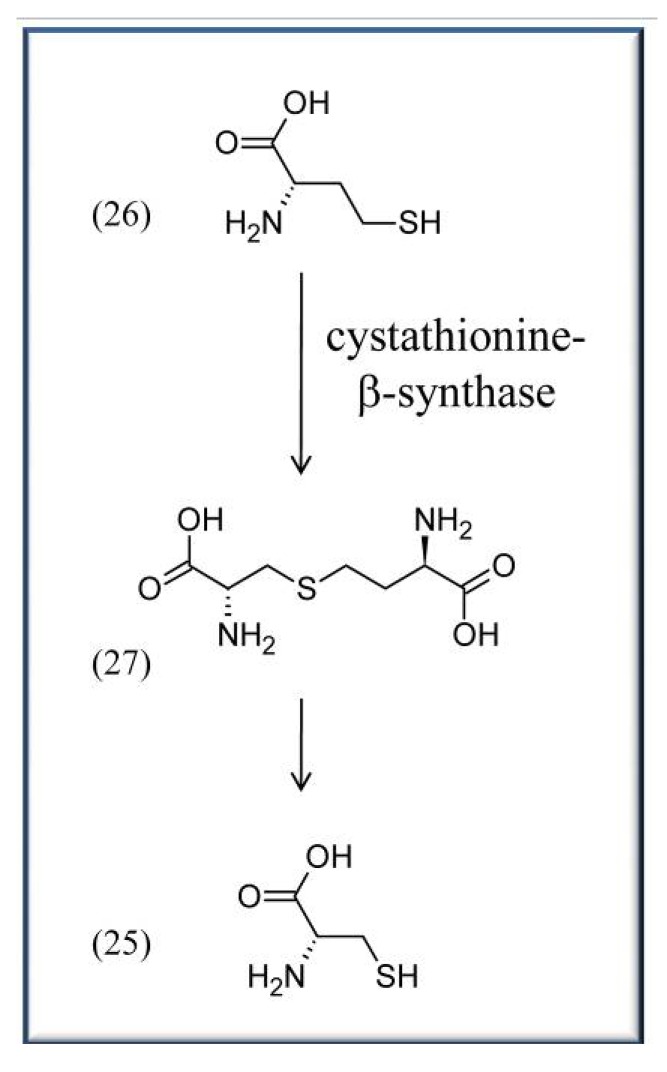
Synthesis of L-cysteine (**25**) from L-homo-cysteine (**26**) *via* the intermediate L-cystathionine (**27**).

Other aspects in which vitamin B_6_ is directly discussed to play an important role are cardiovascular disease and high blood pressure. Coronary heart disease (CHD) is one the major reasons for death worldwide. It is caused by atheromata, which are swollen artery walls due to the accumulation of cell debris containing e.g., fatty acids and cholesterols that negatively affect blood flow. Though the impact of vitB_6_ is controversially discussed (compare for example [52] and [53]), a variety of works indicate positive effects of vitB_6_ on CHD. For instance, a large study in Japan, comprising 40,803 subjects, recently showed that vitB_6_ has the potential to reduce the risk of CHD, and especially nonfatal myocardial infarction (MI), among middle-aged (40–59 years) non-multivitamin supplement users [53]. Here, an increase of daily supplementary vitB_6_ intake from 1.3 to 1.6 mg already significantly reduced the number of affected patients with reported CHDs and MIs [53]. Similarly, the Coronary Health Project and other studies indicate a correlation between increased vitB_6_ intake and reduced risk of CHD [25,54,55,56]. It is noteworthy that often other vitamins like folates or cobalamins are tested in these studies as well with similar positive effects in reducing the risk of CHD. The precise reason(s) for the beneficial impact of vitB_6_ are unclear. One suggested reason is that vitB_6_, like folates and cobalamins, can lower homocysteine **(26)** levels in the blood by converting the amino acid to cysteine (**25**) or methionine, respectively. VitB_6_ is required as a cofactor for cystathionine-β-synthase (EC 4.2.1.22), a PLP-dependent enzyme that converts homocysteine (**26**) to cysteine (**25**) *via* a cystathionine (**27**) intermediate (Scheme 3) [57]. Because high levels of homocysteine are often associated with an increased chance for atherosclerotic diseases, it is considered a risk factor like e.g. high blood pressure, active smoking, or adverse blood lipid profiles [58]. But, as stressed above, it is not generally accepted whether vitB_6_, folates, or cobalamins do indeed reduce the blood homocysteine levels, as a recent review indicates, thus still awaiting additional proof [39].

VitB_6_ appears also to have a beneficial role in reducing hypertension or high blood pressure. Several articles showed that supplementary treatments with the vitamin could lower blood pressure [59,60,61,62,63,64]. Like for CHD the biochemical or physiological reasons are unresolved. However, it is suggested that the blood pressure lowering role of vitB_6_ might be connected with the level of blood aldehydes. These are highly reactive compounds that, potentially by binding to sulfhydryl groups of membrane proteins, activate Ca^2+^-channels and increase cytosolic free calcium in the blood, ultimately leading to an increase in peripheral vascular resistance and blood pressure [65]. Consequently, it is not uncommon for people with excessive alcohol consumption to have increased levels of acetaldehyde, which is often accompanied by high blood pressure [66]. Treatment with *N*-acetylcysteine can normalize blood pressure in spontaneous hypertensive rats, most likely because the amino acid competes with membrane proteins for the reaction with the aldehydes, causing a reduced Ca^2+^ flux [67]. In addition, it is well established that acetaldehyde is detrimental to PLP (**5**) stability [68]. Because PLP (**5**) is needed for the biosynthesis of cysteine (**25**), it is suggested that the mechanism of PLP (**5**) on the blood pressure is either a direct one, by buffering the detrimental activity of aldehydes, or occurs indirectly, by influencing the rate of cysteine (**25**) biosynthesis [61,63]. Still, it is worth mentioning that the connection of PLP (**5**) with lowered blood pressure is curious as it is required for dopamine biosynthesis (see Section 4.3 below), a known vasopressor that actually stimulates contraction of the muscular tissue of the capillaries and arteries [69]. A correlation between high blood pressure and *PDXH/Pnpo* gene expression was found in hypertensive Dahl-S rats [64]. These rats are sensitive to a high salt diet and develop high blood pressure in response to such foods [70]. Okuda and co-workers could show for Dahl-S rats supplied with a high salt diet that, in comparison to a control group, gene expression of the oxidase is down-regulated. These findings indicate that high blood PLP (**5**) levels are needed for coping with salt uptake and argue that the vitamin is beneficial for preventing high blood pressure [64]. However, it would be interesting to know to what extent neurotransmitter production in these rats is affected as well and whether this correlates with the hypertensive status.

**Scheme 4:**
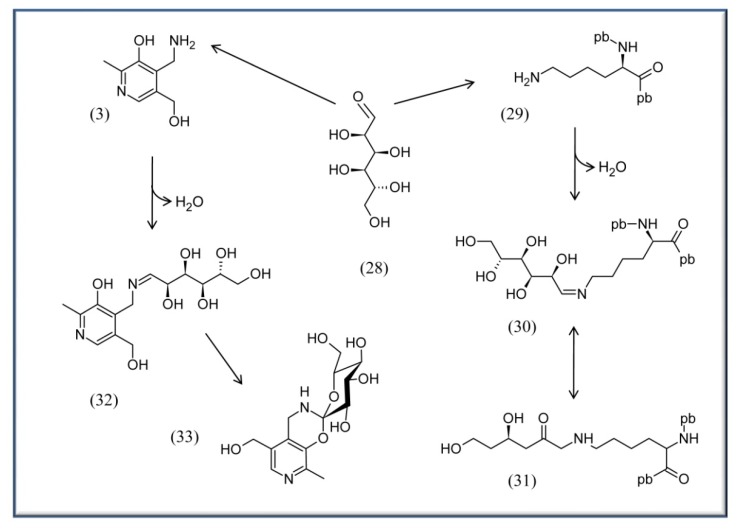
Example for Advanced Glycation Endproduct Reaction of glucose with proteins or PM. On the right half: glucose (**28**) can react with lysine residues of proteins **29** to form an imine (Schiff base) **30** and an Amadori product **31**. This latter can be further oxidized by metals to form the final AGE. pb, peptide bond. On the left half: PM (**3**) has been proven to be the most potent B_6_ vitamer to compete for the formation of AGEs [73]. It is suggested to form first an imine **32** with the glucose, followed by a double cyclization to afford **33** rather than formation of an Amadori product [73].

### 4.3. VitB_6_ in context with diabetes, AGE and ALE

A variety of articles about *diabetes mellitus* focus on the impact of vitB_6_ on blood sugar levels and arteriosclerosis [60,71,72]. For example, a recent study could show that endothelial dysfunction is normalized by treatment with folates and vitB_6_ in children with type 1 diabetes [71]. Endothelial dysfunction is an indicator for the progression of arteriosclerosis that is often developed early in *diabetes mellitus* patients. Endothelial function can be assessed as flow-mediated dilation of the brachial artery with high-resolution ultrasound. MacKenzie and co-workers treated patients over eight weeks with vitB_6_ or folate, which resulted in improved flow-mediated dilation from an average 3.5% to 8.3% and 2.6% to 9.7%, respectively, and with a combination of both to over 10% [71].

Other work also supports the notion of a positive impact of vitB_6_ on endothelial cells, indicating that the vitamin is indeed affecting the status of this tissue [74,75,76]. Additionally vitB_6_ seems to have a positive role against progressive kidney disease, which is frequently associated with diabetic nephropathy [72,77,78]. A possible reason for the advantageous results of vitB_6_ on mammalian tissues is discussed to be the vitamin’s ability to react with reducing sugar and lipids in the blood to prevent formation of advanced glycation or lipoxygenation endproducts (AGE and ALE, respectively) (Scheme 4) [39,72,73,79].

Such products can accumulate when reducing sugars like glucose (**28**) or fructose or polyunsaturated fatty acids are highly abundant in the blood or in cells. This can be the case under stress conditions (e.g. oxidative stress) or for patients that suffer from diabetes or arteriosclerosis, respectively. The accumulation of AGEs and ALEs are on the long run detrimental and can lead to severe tissue damage in the body. Here, vitB_6_ might effectively prevent AGE and ALE formation making it a good candidate as a therapeutic agent in treating side effects in diabetes and arteriosclerosis patients [80,81].

### 4.4. VitB_6_ in context with neurological activity

**Scheme 5:**
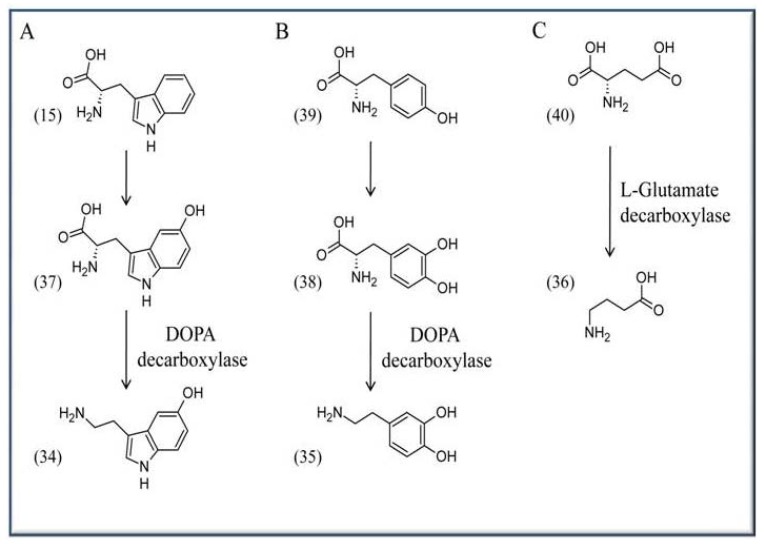
Synthesis of neurotransmitters in the brain. (A) Serotonin (**34**) is synthesized from L-tryptophan (**15**) *via* the intermediate 5-hydroxytryptophan (**37**). (B) Dopamine (**35**) is formed from L-tyrosine (**39**) *via* DOPA (**38**), and (C) GABA (**36**) is formed in a decarboxylation reaction from L-glutamate (**40**).

VitB_6_ is required for the biosynthesis of several neurotransmitters like serotonin (**34**), dopamine (**35**), and γ-aminobutyric acid (GABA) (**36**). Serotonin (**34**), or 5-hydroxytryptamine, is synthesized from L-tryptophan (**15**) and requires the activities of tryptophan hydroxylase (EC 1.14.16.4) and the PLP-dependent enzyme DOPA (L-dihydroxyphenylalanine) decarboxylase [(common synonyms are: L-aromatic amino acid decarboxylase, tryptophan decarboxylase, 5-hydroxytryptophan decarboxylase; (EC 4.1.1.28)], which catalyzes the step from 5-hydroxy-L-tryptophan (**37**) to serotonin (**34**). The enzyme also catalyzes the biosynthesis of dopamine (**35**) from L-DOPA (**38**). Here the initial precursor is L-tyrosine (**37**), which is converted to L-DOPA (**38)** by the activity of L-tyrosine hydroxylase (EC 1.14.16.2) (Scheme 5A, B). GABA (**36**) in turn is synthesized by a decarboxylation reaction from L-glutamate (**40**) based on the activity of L-glutamate decarboxylase (Scheme 5C) (EC 4.1.1.15).

Serotonin (**34**) acts on the central nervous system where it affects a diverse range of conditions including appetite, sleep, or cognitive functions, and it is also well known for its ability to improve the overall mood [82]. In comparison, dopamine (**35**) affects the sympathetic nervous system where it is involved in the regulation of blood pressure and heart rate, while GABA (**36**) is a major inhibitory neurotransmitter in mammals that widely controls the excitability of neurons [83,84]. Consequently, low levels of vitB_6_ have been associated with depression and also dysfunction of the brain (e.g., epilepsy), and it is even considered by some authors as an ‘anti-stress’ agent [85,86,87,88]. In this context it is interesting to note that some plants, like *Ginkgo biloba*, synthesize derivatives of vitB_6_ that are suggested to inhibit the *salvage* pathway enzyme PDXK and thereby to impair neurotransmitter biosynthesis in the brain [8,10,89,90].

### 4.5. The antioxidative capacity of VitB_6_

Only recently was the potent antioxidant ability of vitB_6_ recognized. Here groundbreaking work from the group of Margaret Daub showed that the vitamin is highly efficient in quenching reactive oxygen species with a similar potential like described for carotenes and tocopherols [91,92,93]. Consequently, results from different organisms showed that reduced levels of the vitamin are connected with severe susceptibility to abiotic stress (oxidative, salt, drought, UV-B) [25,93,94,95]. Given the great consideration for other antioxidants like vitamins C and E or phenolics as ‘anti-aging’ compounds by the food industry and consumers, it will be interesting to see whether this relatively novel antioxidant will be embraced in similar ways in the future.

### 4.6. Other aspects related to VitB_6_ and health

VitB_6_ has also been brought into context with various other health aspects. Because these connections between dietary levels of vitB_6_ with disease control are not well established, and could be related to the central or pleiotropic role of vitB_6_ as a cofactor, we will only briefly list some examples that might be of broader interest. For instance, several groups have made connections between high doses of vitB_6_ with reduction of tumor growth potentially by suppressing cell proliferation and angiogenesis [96,97,98,99].

While much of this work has been done in cell cultures, experiments in mice demonstrated significant tumor reduction at the minimum recommended levels of vitB_6_ with optimum reduction at two to five-fold higher levels (up to 35 mg/kg) with no major side effects reported [98]. The immune system depends on vitB_6_ as deficiencies cause ‘atrophy of lymphoid organs, marked reduction in lymphocyte numbers, impaired antibody responses, and decreased IL-2 production’ [100]. Likewise, normal vitB_6_ levels appear to be critical for patients with asthma or carpal tunnel syndrome [101,102]; and finally it appears to be critical for women suffering from premenstrual syndrome (PMS; fatigue, depression, fluid retention etc.) with an apparent correlation between these symptoms and low vitB_6_ levels [103,104].

## 5. Conclusions

The biosynthesis of vitB_6_ and its function as a co-factor have been well resolved in the last years, leaving currently open what drives the activities of the different participating enzymes in the cell. Overall the latest studies indicate that vitB_6_ can be beneficial as a nutritional supplement, but can also be used as a pharmacological agent for disease treatment. Similarly, the diversity of PLP-dependent enzymes and the reactions they catalyze yield a wide range of targets for therapeutic approaches. However, the precise mechanisms of how vitB_6_ is beneficial are often still elusive, and to solidly define them is probably one of the most challenging tasks in the near future.
